# HBFormer: a single-stream framework based on hybrid attention mechanism for identification of human-virus protein–protein interactions

**DOI:** 10.1093/bioinformatics/btae724

**Published:** 2024-12-03

**Authors:** Liyuan Zhang, Sicong Wang, Yadong Wang, Tianyi Zhao

**Affiliations:** School of Computer Science and Technology, Harbin Institute of Technology, Harbin, Heilongjiang 150000, China; Institute of Bioinformatics, Harbin Institute of Technology, Harbin, Heilongjiang 150000, China; School of Computer Science and Technology, Harbin Institute of Technology, Harbin, Heilongjiang 150000, China; School of Computer Science and Technology, Harbin Institute of Technology, Harbin, Heilongjiang 150000, China

## Abstract

**Motivation:**

Exploring human-virus protein–protein interactions (PPIs) is crucial for unraveling the underlying pathogenic mechanisms of viruses. Limitations in the coverage and scalability of high-throughput approaches have impeded the identification of certain key interactions. Current popular computational methods adopt a two-stream pipeline to identify PPIs, which can only achieve relation modeling of protein pairs at the classification phase. However, the fitting capacity of the classifier is insufficient to comprehensively mine the complex interaction patterns between protein pairs.

**Results:**

In this study, we propose a pioneering single-stream framework HBFormer that combines hybrid attention mechanism and multimodal feature fusion strategy for identifying human-virus PPIs. The Transformer architecture based on hybrid attention can bridge the bidirectional information flows between human protein and viral protein, thus unifying joint feature learning and relation modeling of protein pairs. The experimental results demonstrate that HBFormer not only achieves superior performance on multiple human-virus PPI datasets but also outperforms 5 other state-of-the-art human-virus PPI identification methods. Moreover, ablation studies and scalability experiments further validate the effectiveness of our single-stream framework.

**Availability and implementation:**

Codes and datasets are available at https://github.com/RmQ5v/HBFormer.

## 1 Introduction 

Viruses are one of the major pathogenic agents of global infectious diseases. Within the intricate human-virus interaction system, protein–protein interactions (PPIs) are tightly linked with multiple pivotal biological processes underlying viral infection (Dyer *et al.* 2008, Lasso *et al.* 2019). The surface proteins of the virus can bind to specific cellular receptors in human, initiating viral invasion and triggering a series of intracellular signaling cascades, such as mediating cytoskeleton remodeling, activating protein kinases, and suppressing host cell immune response (Grove and Marsh 2011, Dey and Mondal 2024). Also due to the small genome, the virus has to exploit the established functions of human cellular proteins to complete its life cycle (Yang *et al.* 2019). Thus, identifying human-virus PPIs is critical for unraveling the underlying pathogenic mechanisms of viruses and for the development of targeted antiviral therapies.

Affinity purification coupled with mass spectrometry (AP-MS) and the yeast two-hybrid (Y2H) system have been extensively employed in enriching and identifying stable interaction partners of specific proteins (Brückner *et al.* 2009, Qin *et al.* 2021). Moreover, the Y2H system offers the advantage of capturing weak or transient protein interactions in actual cellular processes (Stynen *et al.* 2012). Such finely regulated processes exist for many important signaling proteins such as receptor kinases as well as drivers of cellular genealogy (Xing *et al.* 2014). However, even with substantial investments of time and resources in biological experiments, a great number of false positive results are inevitably generated and many known interactions are frequently missed (Peng *et al.* 2017). Consequently, computational approaches for identifying potential PPIs have been on the rise, providing testable hypotheses that act as a supplement for experimental efforts.

Numerous traditional machine learning approaches have made progress in the identification of PPIs (Yang *et al.* 2010, Cui *et al.* 2012, Dey *et al.* 2020). For instance, Dey *et al.* combined random forest with support vector machine to predict viral-host PPIs on protein datasets characterized by amino acid composition, pseudo amino acid composition, and conjoint triad. Yang *et al.* encoded protein sequence into vectors by local descriptors and leveraged k-nearest neighbor model to detect protein interaction patterns. However, these methods based on hand-crafted feature extraction have limited capabilities in constructing complex hierarchical feature representations, which restrict the classification performance of machine learning models. Given that feature extraction is the key to mining the information patterns of proteins, several tools that integrate protein feature extraction utilities have emerged, such as BioSeq-BLM (Li *et al.* 2021) and iLearnPlus (Chen *et al.* 2021).

In recent years, deep learning techniques have become a promising strategy for accelerating PPI identification research (Yang *et al.* 2022). DPPI (Hashemifar *et al.* 2018) was the first approach to deploy deep learning into identification of PPIs, which designed stacked convolutional neural modules to detect local patterns within sequence profiles, and then performed binary classification through linear layers. After that, PIPR (Chen *et al.* 2019) introduced a residual recurrent convolutional neural network (CNN) (LeCun and Bengio 1995) aimed at providing multi-granular sequence feature aggregation. TransPPI (Yang *et al.* 2021) mined and generated high-dimensional sequence embeddings from sequence profiles based on a Siamese CNN model to facilitate human-virus PPI identification. Nonetheless, dependencies of the global nature of protein sequences are difficult to capture by local convolution operations, and the lack of global features degrades the performance of deep learning approaches. To efficiently model long-distance dependencies of sequences, some works attempted to introduce long short-term memory (LSTM) (Hochreiter and Schmidhuber 1997) neural network. For instance, DNN-PPI (Li *et al.* 2018) constructed LSTM network to capture short-term dependencies at the amino acid level and long-term dependencies at the motif level. LSTM-PHV (Tsukiyama *et al.* 2021) leveraged LSTM with the word embedding model word2vec to learn the context information of amino acid sequences and derived predicted output via fully connected neural networks.

While the mentioned methods have achieved breakthroughs in PPI identification domain, they still suffer from certain limitations. On the one hand, they focus solely on sequence information of proteins, often overlooking other relevant factors acting on PPIs such as biological processes and molecular functions. For instance, post-translational modifications (PTMs) can alter the spatial conformation of proteins, thereby influencing the binding ability to interact with other proteins (Wang *et al.* 2022). On the other hand, existing approaches adopt a two-stream pipeline for PPI identification, which means that separate feature extraction (two-stream) is performed on each of the two input proteins, and then the two sets of feature vectors are concatenated and fed into a classifier to capture the relationships between proteins. However, for different datasets, the input proteins in the two-stream stage may originate from different species, posing a great challenge to the generalization ability of the feature extractor. Moreover, the two-stream framework is constrained to achieving the relation modeling of protein pairs exclusively at the classification phase. Common classifiers essentially aggregate multiple features in a certain proportion of weights, yet this aggregation capability is relatively limited. The fitting capacity of the classifier module is insufficient to comprehensively mine the complex interaction patterns between protein pairs.

To address the above challenges, we propose a single-stream framework named HBFormer, for identifying human-virus PPIs. This predictor is constructed based on hybrid attention mechanism and multimodal feature fusion strategy for proteins. The protein biological annotation features are tokenized to achieve interaction with sequence features in order to comprehensively characterize the intrinsic relationship between protein pairs, improving the prediction performance of the model. Meanwhile, the Transformer with hybrid attention can bridge the bidirectional information flows between human protein and viral protein, thereby unifies joint feature learning and relation modeling of protein pairs. Experimental results indicate that HBFormer outperforms other state-of-the-art methods and significantly impacts human-virus PPI identification.

## 2 Materials and methods

### 2.1 Overview of HBFormer

**Figure 1.**
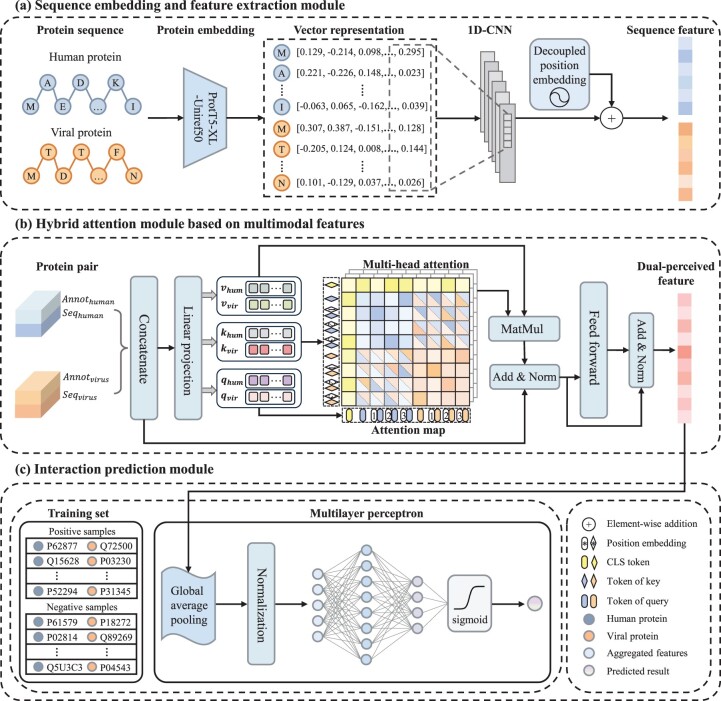
The framework of HBFormer. (a) Sequence embedding and feature extraction module. The pre-trained protein language model ProtT5-XL-Uniref50 and decoupled position encoding are applied to generate informative sequence feature representations of human proteins and viral proteins. (b) Hybrid attention module based on multimodal features. The protein biological annotation features are tokenized to achieve interaction with sequence features to comprehensively characterize the intrinsic relationship between protein pairs. The hybrid attention has dual attention operations, where self-attention allows to learn and extract features from protein itself while cross-attention aims to model the relationship between human protein and viral protein. The dual-perceived features for each of the protein pairs can be obtained in this module. (c) Interaction prediction module. A multilayer perceptron is employed to learn hidden representations from dual-perceived features and calculate predicted probability score of interaction.

As shown in Fig. 1, the HBFormer framework encompasses three main parts: sequence embedding and feature extraction module, hybrid attention module based on multimodal features and interaction prediction module.

### 2.2 Sequence feature representation

Pre-trained protein language models are a powerful paradigm for creating effective embeddings and learning context-aware data representations and have been successfully used in various downstream protein understanding tasks (Liu and Tian 2023, Wang *et al.* 2023). In this study, we adopt ProtT5-XL-Uniref50 (PT5) (Elnaggar *et al.* 2022) to embed human proteins and viral proteins for more informative sequence representations. PT5 is a pre-trained model based on the Text-to-Text Transfer Transformer (T5) (Raffel *et al.* 2020) architecture, conducted in a self-supervised manner through masked language modeling. This model is not only capable of capturing complex pattern in sequence contexts, but also generating different embedding representations of the same amino acid according to different contextual semantic information. It comprises a 24-layer encoder–decoder, encompassing 3B parameters. The pretext task of PT5 is to reconstruct inputs from masked tokens (amino acids), aiming at training a more powerful feature encoder. The whole process is trained on the BFD (Steinegger and Söding 2018) protein sequence corpora, which contain 393 billion amino acids, and then fine-tuned on the UniRef50 (Suzek *et al.* 2015) dataset. In implementation, we freeze the weights of the fine-tuned PT5 model to encode sequence. Each amino acid in the human and viral protein sequence is output as a 1024-dimensional embedding vector.

For each human protein sequence embedded by PT5, we denote it as $\left[a_{h}^{1},a_{h}^{2},a_{h}^{3},\ldots ,a_{h}^{m}\right]$, similarly, each viral protein sequence can be described as $\left[a_{v}^{1},a_{v}^{2},a_{v}^{3},\ldots ,a_{v}^{n}\right]$, where *m* and *n* are the lengths of the human and viral protein sequences, respectively, and *a* represents the encodings of amino acid. Next, CNNs with shared weights are utilized to project the sequence embeddings of the human and viral proteins from the initial dimension into the *D* dimension latent space. Meanwhile, we construct decoupled learnable positional encodings *P_h_* and *P_v_*, and separately added them to the corresponding embeddings. This step is necessitated as the subsequent attention mechanism is essentially an order-independent set operation, so explicit position encoding is required to preserve and distinguish the information of amino acid residues at different positions. Moreover, considering the variable lengths of different protein sequences, the proximal interpolation is incorporated to extend the learnable positional embedding matrix along the sequence dimension *N* to match the specific length of each protein sequence. The final sequence feature representations of human protein and viral protein are as follows:

(1) $${\text{Seq}}_{\text{hum}}={\text{Conv}}_{1D}[a_{h}^{1};a_{h}^{2};\ldots ;a_{h}^{m}]+\tau (P_{h}),P_{h}\in R^{N_{h}\times D},$$

(2) $${\text{Seq}}_{\text{vir}}={\text{Conv}}_{1D}[a_{v}^{1};a_{v}^{2};\ldots ;a_{v}^{n}]+\tau (P_{v}),P_{v}\in R^{N_{v}\times D},$$

where τ(·) is the proximal interpolation and *D* is set to 360.

### 2.3 Biological annotation feature representation

In this section, we firstly employ one-hot encoding to convert biological annotation information into numerical features. The biological annotation data for human protein and viral protein is collected from the UniProt (Consortium 2019) database, which contains entries based on six major categories of keywords, including biological process, cellular component, disease, domain, molecular function, and PTM. Assuming that there are total *k* entries of human proteins and viral proteins, then the biological annotation feature of proteins can be denoted as follows:

(3) $$\text{Annot}=[e_{1},e_{2},e_{3},\ldots ,e_{k}],$$

where *e_i_* represents the *i*th protein biological annotation property. If a human or viral protein has this property, *e_i_*=1, otherwise, *e_i_*=0.

Due to the large number of entries encompassed by the biological annotation data, the dimensionality of features is correspondingly high. Consequently, principal component analysis is introduced to reduce the dimensionality of the biological annotation features and to mitigate potential noise effects. We preserve 98% of the biological annotation feature information for both human proteins and viral proteins.

### 2.4 Transformer based on hybrid attention mechanism with multimodal features

Attention module is a highly flexible architectural component with dynamic and global modeling capacity, which has been successfully applied across diverse domains, including natural language processing and image recognition (Vaswani *et al.* 2017, Dosovitskiy *et al.* 2021). Drawing inspiration from this, we propose a single-stream Transformer framework, the core objective of which is to leverage the hybrid attention mechanism to bridge the bidirectional information flows between human proteins and viral proteins while simultaneously performing feature extraction and relation modeling. Herein, the hybrid attention has dual attention operations, where self-attention allows to learn and extract features from protein itself while cross-attention aims to model the mutual interaction patterns between human protein and viral protein. We will demonstrate the computational process of the hybrid attention module through mathematical derivation and analyze its intrinsic reasons. The input of the hybrid attention module is the concatenation of *F*_hum_, *F*_vir_, and *CLS* token, denoted as: [*F*_hum_; *F*_vir_; *CLS*], where *F*_hum_ is the concatenation of sequence feature and biological annotation feature of human protein, and *F*_vir_ is analogous to it. The *CLS* token refers to the class token, a special learnable token that serves to aggregate information from other tokens, providing the global feature representation. For simplicity, we omit the class token in the derivations. The output of the attention operation can be calculated as:

(4) $$\begin{array}{c} \text{Attention}(Q,K,V)=\text{Softmax}\left(\frac{QK^{T}}{\sqrt{d_{k}}}\right)\cdot V\\ =\text{Softmax}\left(\frac{[Q_{\text{vir}};Q_{\text{hum}}]{\left[K_{\text{vir}};K_{\text{hum}}\right]}^{T}}{\sqrt{d_{k}}}\right)\cdot [V_{\text{vir}};V_{\text{hum}}], \end{array}$$

where *Q*, *K*, and *V* denote query, key, and value matrices, respectively. The subscripts hum and vir denote the matrix items representing the human protein and the viral protein. Next, we expand the attention weights in Equation (4), which is calculated as follows:

(5) $$\begin{array}{c} \text{Softmax}\left(\frac{[Q_{\text{vir}};Q_{\text{hum}}]{\left[K_{\text{vir}};K_{\text{hum}}\right]}^{T}}{\sqrt{d_{k}}}\right)\\ =\text{Softmax}\left(\frac{\left[Q_{\text{vir}}K_{\text{vir}}^{T},Q_{\text{vir}}K_{\text{hum}}^{T};Q_{\text{hum}}K_{\text{vir}}^{T},Q_{\text{hum}}K_{\text{hum}}^{T}\right]}{\sqrt{d_{k}}}\right)\\ ≜[Y_{\text{vir}\_\text{vir}},Y_{\text{vir}\_\text{hum}};Y_{\text{hum}\_\text{vir}},Y_{\text{hum}\_\text{hum}}], \end{array}$$

where $Y_{\text{vir}\_\text{hum}}$ and $Y_{\text{hum}\_\text{vir}}$ denote the weights that quantifies mutual relation patterns between human protein and viral protein (relation modeling), while $Y_{\text{vir}\_\text{vir}}$ and $Y_{\text{hum}\_\text{hum}}$ represent the weights that aggregate the protein features (feature extraction). It needs to be noted that the attention mechanism dynamically assigns weights during information aggregation. The more critical the features affecting the interactions, the higher the corresponding weights in the attention map. Then, we can calculate the output as follows:

(6) $$\begin{array}{c} \text{Attention}(Q,K,V)=[Y_{\text{vir}\_\text{vir}}V_{\text{vir}}+Y_{\text{vir}\_\text{hum}}V_{\text{hum}};\\ Y_{\text{hum}\_\text{vir}}V_{\text{vir}}+Y_{\text{hum}\_\text{hum}}V_{\text{hum}}]. \end{array}$$

In the above process, we extend the attention mechanism to the multiple attention heads, which enables it to consider various attention distributions and allows the model to focus on different aspects of protein multimodal features.

(7) $$\begin{array}{c} \text{MultiHead}\;(Q,K,V)=\text{Concat}\;({\text{head}}_{1},\ldots ,{\text{head}}_{h})W^{O}\\ {\text{head}}_{i}=\text{Attention}\;(QW_{i}^{Q},KW_{i}^{K},VW_{i}^{V}), \end{array}$$

where *W^Q^*, *W^K^*, *W^V^*, and *W^O^* are learnable linear projection parameter matrices, the number of attention heads *h* is set to 12. After the attention operation, the output of the multi-head attention mechanism denoted as *Z* is processed as follows:

(8) $$\hat{X}=X+Z,$$

(9) $$\tilde{X}=\text{FFN}\left(\text{LN}\left(\hat{X}\right)\right),$$

(10) $$Z\prime =\text{LN}\left(\tilde{X}+\text{LN}\left(\hat{X}\right)\right),$$

where FFN(·) and LN(·) denotes feed forward layer and layer normalization, respectively, and *X* is [*F*_hum_; *F*_vir_; *CLS*]. Ultimately, we can get the dual-perceived feature Z′ for human-virus protein pairs.

### 2.5 Prediction by multi-layer perceptron

After extracting the dual-perceived features for protein pairs using the hybrid attention mechanism, we construct the prediction module based on a multilayer perceptron. The probability score output from the last fully connected layer is utilized to assess the authenticity of interactions between proteins. The mathematical representation of the multilayer perceptron is:

(11) Z″=LN(Flatten(GAP(Z′))),

(12) $$f_{1}=\sigma_{1}\left(W_{1}\left(Z\prime \prime \right)+b_{1}\right),$$

(13) $$f_{2}=\sigma_{2}\left(W_{2}\left(f_{1}\right)+b_{2}\right),$$

where GAP(·) is the global average pooling operation employed for providing compact feature representations, and Flatten(·) denotes the flatten operator. For the *i*th layer, *W_i_* denotes the weight matrix, *b_i_* is bias vector and *σ_i_* represents the activation function. In our study, *σ*_1_ is the GELU function, and $\sigma_{2}(x_{i})=1/(1+e^{-x_{i}})$ is the sigmoid function. Furthermore, there is an extremely imbalanced distribution of positive and negative samples in the dataset, which may potentially lead to biased model predictions, inundating the predictor with the majority class. To alleviate the problem, we introduce the Focal Loss (Lin *et al.* 2017) to supervise the training of the model. As an improvement of binary cross entropy, its core idea is to down-weight the loss assigned to easy classified examples during training, while focusing more on the hard-to-classify examples. It helps to enhance the model’s capability to discriminate between categories, thus ensuring a more robust and reliable prediction process. The focal loss function is defined as:

(14) $${\text{Loss}}_{\text{focal}}=-\alpha_{t}{\left(1-p_{t}\right)}^{\gamma }\;\text{log}(p_{t}),$$

where *p_t_* represents the predicted probability of the model for the correct class, $\alpha_{t}\in (0,1)$ denotes the balancing factor for adjusting the importance of positive and negative samples, and γ>0 is the focusing parameter to down-weight the easy examples.

## 3 Results

### 3.1 Dataset

- Benchmark dataset
   To measure the performance of HBFormer for identifying human-virus PPIs, we adopt the high-quality benchmark dataset assembled by Tsukiyama *et al.* (2021). The positive samples contain experimentally validated 22 383 PPIs involving in 5882 human proteins and 996 viral proteins. Among the data, the confidence score of each interaction is greater than 0.3. Meanwhile, all proteins are non-redundant, composed of standard amino acids, and had lengths between 30 and 1000 residues. Regarding the construction of negative samples, the dissimilarity negative sampling method (Eid *et al.* 2016) is utilized to effectively mitigate the impact of noise samples that are similar to positive samples. The ratio of positive and negative samples in this benchmark dataset is 1:10. All samples are divided into a training set (conducting cross-validation) and an independent test set with a ratio of 8:2.
- Specific types of human-virus PPI dataset
   In order to comprehensively evaluate the performance of HBFormer, we use the dataset from the TransPPI work (Yang *et al.* 2021). The TransPPI dataset contains experimentally validated information on the interactions between human proteins and proteins of eight specific types of viruses, which are HIV, Herpes, Papilloma, Influenza, Hepatitis, Dengue, Zika, and SARS-CoV-2. The number of positive samples is 9880, 5966, 5099, 3044, 1300, 927, 709, and 568, respectively. Similarly, the negative samples of this dataset are obtained by the dissimilarity negative sampling method with a ratio of positive to negative samples of 1:10.
- Non-viral pathogen PPI dataset
   To investigate the applicability of our method to other types of pathogens, the interaction dataset is taken from Kösesoy *et al.* (2019), which includes PPIs between human and Bacillus anthracis, as well as human and Yersinia pestis. In the Human-Bacillus anthracis PPI dataset, the number of positive and negative interactions is 3090 and 9500, respectively. The Human-Yersinia pestis PPI dataset consists of 4097 positive samples and 12 500 negative samples. Negative samples for both datasets are generated using the random sampling method.

### 3.2 Comparison with state-of-the-art methods

To verify the effectiveness of our model, we compare HBFormer with five other state-of-the-art human-virus PPI identification methods on the benchmark dataset. As shown in Table 1, HBFormer based on the multi-feature fusion strategy surpasses other competing methods across all metrics. When HBFormer solely uses sequence features, it also exhibits superior performance in AUC and AUPRC metrics compared to other approaches, and the higher AUPRC value demonstrates the effectiveness of our single-stream framework in identifying positive examples. We can also observe that the AUPRC of HBFormer is 23.5% higher than that of the DeepViral method (Liu-Wei *et al.* 2021) with the multi-feature fusion strategy, as well as showing higher values in terms of MCC and F1 scores, which can provide better predictions when faced with an imbalance of data. Moreover, HBFormer significantly outperforms the sequence-based identification approaches (Yang *et al.* 2020, Tsukiyama *et al.* 2021, Yang *et al.* 2021, Madan *et al.* 2022), achieving improvements of 4.4%, 4.9%, 5.3%, and 18.2% on AUPRC, which may be attributed to the following key factors: On the one hand, we incorporate comprehensive protein characterization, encompassing not only sequences but also multifaceted biological annotation information, which provides the model with multidimensional insights into proteins. On the other hand, our pioneering single-stream framework is capable of performing additional and enhanced relation modeling between human protein and viral protein prior to classification, thus improving the model’s ability to discriminate interaction patterns.

**Table 1.** Comparison of different methods on benchmark dataset.

Method	AUC	AUPRC	ACC	F1	MCC
DeepViral	0.981	0.757	0.952	0.688	0.674
Yang	0.963	0.810	0.947	0.724	0.697
LSTM-PHV	0.976	0.939	0.984	0.910	0.903
TransPPI	0.982	0.943	0.984	0.896	0.886
STEP	0.983	0.948	0.983	0.908	0.902
HBFormer^a^	0.985	0.948	0.984	0.907	0.898
HBFormer^b^	**0.998**	**0.992**	**0.993**	**0.962**	**0.958**

a Based on single-modal (sequence) features.

b Based on multimodal (sequence+biological annotation) features.

### 3.3 Comparison across specific types of PPI datasets

To further assess the predictive ability of HBFormer across specific types of human-virus PPI datasets, we compare HBFormer with three other advanced identification methods that achieved high performance on the benchmark dataset. The results of the 5-fold cross-validation are shown in Table 2. It can be observed that the AUC metrics of our model show superiority over other advanced methods across all types of human-virus PPI datasets, especially on datasets with smaller data sizes (Hepatitis, Dengue, Zika virus, and SARS-CoV-2). Moreover, except for the slightly lower AUPRC on the Herpes and SARS-CoV-2 datasets compared with the STEP method (Madan *et al.* 2022), HBFormer achieves the highest AUPRC value on the other six datasets. These results imply that our single-stream model exhibits robust classification performance and reliability in uncovering interaction patterns, which is of great importance for advancing the identification of human-virus PPIs.

**Table 2.** Comparison results across specific types of human-virus PPI dataset.

Dataset	Method	AUC	AUPRC	ACC	Dataset	Method	AUC	AUPRC	ACC
Human-HIV	TransPPI	0.995	0.974	0.986	Human-Hepatitis	TransPPI	0.917	0.636	0.934
	LSTM-PHV	0.994	0.959	0.981		LSTM-PHV	0.908	0.559	0.920
	STEP	0.996	0.976	0.988		STEP	0.901	0.639	0.926
	HBformer	**0.996**	**0.976**	**0.989**		HBformer	**0.935**	**0.685**	**0.940**
Human-Herpes	TransPPI	0.941	0.768	0.952	Human-Dengue	TransPPI	0.917	0.636	0.934
	LSTM-PHV	0.936	0.722	0.935		LSTM-PHV	0.891	0.457	0.905
	STEP	0.951	**0.802**	**0.956**		STEP	0.924	0.638	0.933
	HBformer	**0.961**	0.781	0.954		HBformer	**0.958**	**0.683**	**0.940**
Human-Papilloma	TransPPI	0.959	0.818	0.959	Human-Zika	TransPPI	0.926	0.746	**0.954**
	LSTM-PHV	0.954	0.749	0.942		LSTM-PHV	0.867	0.571	0.924
	STEP	0.962	0.823	0.957		STEP	0.937	0.757	0.949
	HBformer	**0.975**	**0.835**	**0.961**		HBformer	**0.951**	**0.802**	0.953
Human-Influenza	TransPPI	0.964	0.834	0.961	Human-SARS-CoV-2	TransPPI	0.805	0.329	0.906
	LSTM-PHV	0.960	0.781	0.948		LSTM-PHV	0.776	0.253	0.869
	STEP	0.959	0.843	0.958		STEP	0.837	**0.428**	**0.911**
	HBformer	**0.965**	**0.849**	**0.962**		HBformer	**0.879**	0.424	0.895

### 3.4 Comparison with popular embedding methods

To probe the capacity of different embedding methods for characterizing protein sequences, we train our model using four other widely used encoding schemes. Note that we construct HBFormer solely based on sequence features to ensure unbiased evaluation. For the static embedding methods Word2vec (Mikolov *et al.* 2013) and FastText (Joulin *et al.* 2016), we additionally fine-tune these models by using the genism library for a more comprehensive comparison. The results in Table 3 demonstrate that HBFormer with PT5 embedding exhibits improved performance compared with other encoding approaches. Specifically, without any fine-tuning on the benchmark dataset, PT5 shows a significant advantage compared with Word2vec and FastText in terms of the AUPRC. We also observe that these static embedding methods can lead to a significant improvement in the performance of classification models after fine-tuning on the dataset. However, their performance is still inferior to the PT5 method based on self-supervised training. This is attributed to PT5’s ability to learn context-specific sequence semantic information, enabling the generation of robust expressions enriched with more global contextual information and long-distance dependencies. In contrast to the pre-trained protein language model ESM-1B (Rives *et al.* 2021), which is also based on self-supervised training, PT5 presents superior performance across all metrics, indicating it can achieve more informative sequence representations. Furthermore, the AUC and AUPRC of PT5 are 2.9% and 12.7% higher than those of the method Bepler (Bepler and Berger 2019), which utilizes global structural similarity between proteins for weakly supervised embedding training, further illustrating the powerful expressive capabilities of sequence features derived from PT5.

**Table 3.** Comparison results of different embedding methods.

Methods	AUC	AUPRC	ACC	F1	MCC
Word2vec	0.890	0.519	0.889	0.526	0.482
Word2vec^a^	0.971	0.926	0.978	0.878	0.867
FastText	0.907	0.580	0.889	0.550	0.515
FastText^a^	0.974	0.924	0.979	0.873	0.861
ESM-1B	0.949	0.761	0.924	0.656	0.639
Bepler	0.956	0.821	0.944	0.726	0.700
PT5	**0.985**	**0.948**	**0.984**	**0.907**	**0.898**

a Fine-tuned on the benchmark dataset.

### 3.5 Ablation study

**Figure 2.**
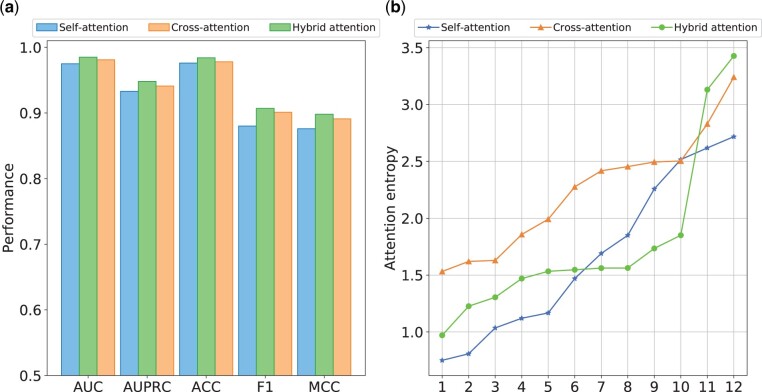
Ablation studies. (a) Performance comparison using different attention mechanism. (b) Attention entropy distribution across all attention heads using different attention mechanism.

To investigate the contribution of the hybrid attention mechanism, we test the performance of HBFormer with its self-attention module or cross-attention module removed. In this section, we train the HBFormer only using sequence features to eliminate potential biases introduced by biological annotation features. The results of the ablation study conducted on the independent test set are shown in Fig. 2a. It can be observed that the performance of HBFormer seems to decrease when we remove any part of the attention components. This elucidates that both self-attention and cross-attention contribute to the identification of interactions, but the impact on the model’s performance is greater in the absence of cross-attention, which may be due to the fact that cross-attention can provide additional relationship modeling and thus more effectively mine the relationships between proteins. Notably, HBFormer can be categorized as a two-stream framework when utilizing only self-attention, as in this stage, the Transformer only amounts to separate feature extraction operations for each of the two proteins. This further suggests that the single-stream attention mechanism framework outperforms the two-stream framework in the PPI identification tasks.

We further perform an analysis of the information aggregation capabilities of different attention modules. Specifically, we introduce the attention entropy metric to evaluate the attention maps, where the attention maps are calculated from the compatibility of queries and keys via dot-product operations. The attention entropy is defined as follows:

(15) $$E_{h,j}=-\sum_{i=1}^{l}\text{softmax}{\left(A_{h}\right)}_{i,j}\;\text{log}(\text{softmax}{\left(A_{h}\right)}_{i,j}),$$

where *A_h_* is the attention map for the *h_th_* attention head, *i*, *j* represent the *i*th and *j*th tokens, *l* is the number of tokens and $\text{softmax}(x_{i})=e^{x_{i}}/\sum_{j}e^{x_{j}}$. For the *h_th_* head, the average attention entropy across all tokens can be calculated as:

(16) $$e_{h}=\frac{1}{l}\sum_{j=1}^{l}E_{h,j}.$$

In this study, we use a total of 12 heads based on multi-head attention mechanism. The average attention entropy distribution across all attention heads is shown in Fig. 2b. The HBFormer based on the hybrid attention mechanism exhibits a broader distribution of attention entropy compared with models employing only self-attention or cross-attention, which means that the attention heads have different specialization roles, allowing the model to better aggregate both local and global tokens with both concentrated and broad focuses. The above results prove that the hybrid attention mechanism is indispensable in our single-stream framework, facilitating the discrimination between interacting and non-interacting protein pairs.

### 3.6 Identification of non-viral pathogens PPIs

In order to further assess the generalizability of our proposed framework, we apply HBFormer to identify the interactions of human proteins with other non-viral pathogen proteins. We perform 5-fold cross-validation on the PPI datasets of Human-Bacillus anthracis (Human-B) and Humans-Yersinia pestis (Human-Y), respectively, and average the performance metrics of the subset models to produce more reliable predictions. The results are presented in Table 4. HBFormer still shows commendable performance on the two cross-species datasets, with AUC, AUPRC, and ACC all reaching above 0.9, suggesting that HBFormer can also be effectively extended to the PPI identification in humans and other types of pathogens.

**Table 4.** Results on non-viral pathogens datasets.

Dataset	AUC	AUPRC	ACC	F1	MCC
Human-B	0.973	0.928	0.949	0.874	0.825
Humans-Y	0.961	0.909	0.935	0.853	0.792

## 4 Discussion

In this paper, wek propose HBFormer, a single-stream computational framework based on the hybrid attention mechanism for identifying human-virus PPIs. Unlike other deep learning approaches for PPI identification, our pioneering single-stream framework is capable of achieving additional relation modeling between human protein and viral protein simultaneously with feature extraction, thereby improving the model’s ability to discriminate interaction patterns. Moreover, the multimodal feature fusion strategy adopted is also an important factor contributing to the excellent performance of HBFormer, which provides the model with both comprehensive and multidimensional insights into proteins. Extensive experiments validate the effectiveness of our proposed framework and demonstrate its extensibility to other types of PPI identification. However, there remain some shortcomings in this research. The imbalance learning strategy we incorporate may still not fully resolve the problem of skewed sample distributions in some categories, and thus more ways to rectify the imbalance still need to be explored in order to improve the performance of the model. Furthermore, due to the high computation and memory demands of the Transformer architecture, insufficient computational resources may affect the application of the model. Optimizing computation to break through speed bottlenecks with FlashAttention tools will be an extensible work in the future.

Conflict of interest: The authors declare no competing interests.

## Data Availability

All data and source code used in the present study is available at https://github.com/RmQ5v/HBFormer.
